# From service design thinking to the third generation of activity theory: a new model for designing AI-based decision-support systems

**DOI:** 10.3389/frai.2024.1303691

**Published:** 2024-03-21

**Authors:** Silvia Marocco, Alessandra Talamo, Francesca Quintiliani

**Affiliations:** ^1^Department of Social and Developmental Psychology, Sapienza University of Rome, Rome, Italy; ^2^Mylia – Adecco Formazione S.r.l., Milan, Italy

**Keywords:** activity theory, service design thinking, multi-actor decision-making, investments in human capital, organizational development, decision-support systems

## Abstract

**Introduction:**

The rise of Artificial Intelligence (AI), particularly machine learning, has brought a significant transformation in decision-making (DM) processes within organizations, with AI gradually assuming responsibilities that were traditionally performed by humans. However, as shown by recent findings, the acceptance of AI-based solutions in DM remains a concern as individuals still strongly prefer human intervention. This resistance can be attributed to psychological factors and other trust-related issues. To address these challenges, recent studies show that practical guidelines for user-centered design of AI are needed to promote justified trust in AI-based systems.

**Methods and results:**

To this aim, our study bridges Service Design Thinking and the third generation of Activity Theory to create a model which serves as a set of practical guidelines for the user centered design of Multi-Actor AI-based DSS. This model is created through the qualitative study of human activity as a unit of analysis. Nevertheless, it holds the potential for further enhancement through the application of quantitative methods to explore its diverse dimensions more extensively. As an illustrative example, we used a case study in the field of human capital investments, with a particular focus on organizational development, which involves managers, professionals, coaches and other significant actors. As a result, the qualitative methodology employed in our study can be characterized as a “pre-quantitative” investigation.

**Discussion:**

This framework aims at locating the contribution of AI in complex human activity and identifying the potential role of quantitative data in it.

## Introduction

The rise of Artificial Intelligence (AI), particularly machine learning, has brought a significant transformation in decision-making (DM) processes within organizations, with AI gradually assuming responsibilities that were traditionally performed by humans (Vincent, 2021). The selection of AI approaches in DM has been traditionally determined by the nature of the task, with routine and well-structured tasks being automated and more complex tasks being addressed through augmentation, which emphasizes a supportive role of AI rather than a complete substitution. In this context, Decision Support Systems (DSS) have emerged as crucial tools in aiding organizational members across various activities, including planning and operational execution (Gupta et al., 2020). As DSS integrate AI technology, they demonstrate adaptability and organizational capabilities within uncertain and dynamic environments (Keith and Ahner, 2019). However, as shown by recent findings, the acceptance of AI-based solutions in DM remains a concern as individuals still strongly prefer human intervention (Haesevoets et al., 2021). This algorithm resistance can be attributed to various barriers, including psychological factors and other trust-related issues (Cao et al., 2021; Leyer and Schneider, 2021; Mahmud et al., 2023). To address these challenges, recent studies demonstrate that practical guidelines for user-centered design are necessary to foster justified trust in AI-based systems (Shin, 2021). This approach focuses on understanding and addressing the needs, preferences, and concerns of the prospective users, placing them at the center of the design process.

While previous research on human-machine interaction has primarily focused on the external side of user experience, such as interface design and usability, we recognize that the internal processes of AI technologies also require to be designed according to a human-centered perspective (Talamo et al., 2021). Indeed, the context in which AI systems are employed, the practical actions and circumstances involved, and the attitudes of humans towards artificial agents all shape the effectiveness and acceptance of these tools (Heath and Luff, 2000). As a result, it becomes crucial to move beyond a purely technical perspective and consider the social and contextual aspects of technological integration (Suchman, 1987). This entails focusing on the analysis of contextualized human reasoning models to shape the internal processes of AI technologies.

Moreover, psychological research has been extensively applied to study various psychological phenomena in DM using quantitative tools. However, this approach has presented several limitations, including an imbalance in the prevalence of studies, with a focus on analyzing individual DM processes, or the frequent perception of humans as carriers of biases and distortions (Marocco and Talamo, 2022). Hence, we recognized the need for a different approach, that views activity systems as the primary unit of analysis.

To address these needs, Activity Theory (AT), pioneered by Leont’ev (1974, 1978), can be adopted. Indeed, this theory enables to capture the crucial aspects of actual technology usage and the descriptive and generalizable qualities requisite for practicality and efficacy in the context of Interaction Design (Kaptelinin and Nardi, 2006). There is also growing evidence of the relevance of including ecological criteria for designing technologies (Talamo et al., 2011), to capture the complexity and contingency of real-life actions in specific situations (Talamo et al., 2015).

Furthermore, AT provides a valuable framework for modeling the complexities of DM processes. More specifically, by adopting the third generation of AT (Engeström, 1987, 2001), which emphasizes dialogue, multiple perspectives, and networks of interacting activity systems, it is possible to gain insights into the intricacies of Multi-Actor DM (MADM), where nor a single group with a shared goal is engaged in the DM process, but multiple individuals, groups, or organizations that, starting from not-coinciding objectives, interact with each other through a negotiation process to reach a mutual agreement and converge towards a common goal for the success of the investment (Marocco and Talamo, 2022). Additionally, the holistic nature of the service surrounding the tool plays a vital role. There is already a significant tradition in literature that approaches Information Technologies (IT) Design by analyzing the decomposition, matching, and discovery of services, known as Service-Oriented Architecture (SOA). In SOA, software resources are packaged as “services,” which are autonomous and well-defined modules providing standard business functionalities and are independent of the state or context of other services (Papazoglou and Heuvel, 2007). However, in our study, we chose Service Design Thinking (SDT), a multidisciplinary approach that combines methods from various disciplines to create or enhance experiences or services (Stickdorn and Schneider, 2011), to emphasize human-centered contextualization from a psychological perspective. Moreover, with respect to SOA Design, this approach proves beneficial for discerning which activities ought to be augmented by AI and which should stay under the purview of human-beings.

This paper aims to explore and propose practical guidelines for enhancing the user-centered design of Multi-Actor AI-based DSS. In particular, our aim is to develop the overall underlying service in which AI will be integrated. As an illustrative example, in this paper we used a case study in the field of *investments in human capital* (IHC), with a particular focus on personnel’s organizational development. This context is characterized by the specificity of MADM. In fact, at least two distinct investment decisions made by multiple actors can be highlighted. From one perspective, the HR manager and the People Manager, acting as investors, face the task of deciding whether company resources should be allocated to support an employee’s developmental journey. On the other hand, the employee, seeking personal investment, reflects on whether dedicating their time and effort to a company-proposed developmental path aligns with their individual goals and motivations.

Starting from the SDT process and leveraging the potential of AT, we propose a holistic model—the MADM model—for the design of Multi-Actor AI-based DSS in the context of organizational development. The following sections will delve into the theoretical foundations, research methodology, and practical instructions for the definition of the MADM model.

## Modeling human MADM: the contribution of activity theory

**Figure 1:**
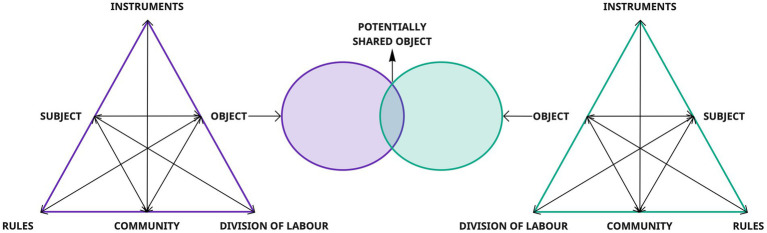
III generation of AT (Engeström, 2001).

Due to the complex and multi-layered nature of DM in the field of IHC (Marocco and Talamo, 2022), we believe it is more beneficial to adopt the third generation of AT as proposed by Engeström in 2001 (Figure 1). This expansion specifically addresses the challenge of developing *“conceptual tools to understand dialogue, multiple perspectives, and networks of interacting activity systems”* (Engeström, 2001, p. 135). Indeed, if with the second generation of AT, Engeström introduced the concept of the *activity system*, expanding upon the *Subject-Artifact-Object* triangle (Leont’ev, 1974, 1978) with three new elements (*rules*,^1^ *division of labor*,^2^ and *community*;^3^) through the third generation of AT, Engeström emphasizes the interconnections among different activity systems that produce a multiplicity of voices and interact with their partially *shared objects*. Furthermore, the core of this theoretical reconceptualization regards the concept of *object*, which is defined by Engeström as “*a project under construction, moving from potential raw material to a meaningful shape and to a result or outcome”* and as what *“determines the horizon of possible goals and actions”* (Engeström, 1999, p. 65). Moreover, in the third generation of AT, Engeström describes the object as a potentially shared or jointly constructed object. This is particularly important when considering organizational analysis, since organizations center their activities around objects that are “*partly shared, partly fragmented, possibly contested, and certainly emergent, and because objects of activity are likely to be rooted in multiple activity systems, they may not be at all easy to change in the short term”* (Sannino et al., 2009, p. 27). This means that across multiple activity systems, there can be shared horizons of specific goals and actions. The object, indeed, serves as a point of convergence, where different activity systems may align their objectives and actions towards a common purpose.

For this reason, we propose the third-generation of AT (Figure 1) (Engeström, 2001) as a model to explain the specificities of the MADM construct, in a way to physically visualize the specificity of each activity system included in the DM process, including the specific goals of the subjects (decision-makers) and how they converge to create a potentially *shared object*.

### The creation of interobjectivity between activity systems

When multiple activity systems share a common orientation towards the same object, this can lead to the creation of *interobjectivity*. Moghaddam (2003) introduced the concept of *interobjectivity*, referring to two distinct levels of analysis. Firstly, within groups, interobjectivity describes the shared meanings and understandings of objective reality that individuals have within the same cultural context. It highlights how people within a group develop a common understanding of certain objects. Secondly, between groups, interobjectivity refers to the representation of an object that incorporates diverse social meanings existing among different cultural groups. According to Moghaddam (2003, p. 230), it is practical experiences that lead different individuals or groups to recognize that *“…through the* var*ious collaborative tasks (…) it is possible to understand others, and for them to understand us.”* Therefore, the concept of interobjectivity directs attention towards the collaboratively constructed world outside individuals and views subjective understandings as emerging from participation in collective processes. As a result, Talamo and Pozzi (2011, p. 304), building upon Moghaddam’s (2003) definition, interpret interobjectivity as *“the common orientation of participants towards a practical goal and as the process by which a practical activity is jointly undertaken by different subjects.”* Consequently, objects that belong to multiple activity systems, as in the case of MADM, require analytical work to identify the various points of convergence that enable their compatibility and potential sharing. This approach seeks to verify how specific objectives of the subjects involved may align towards a shared and unified vision: the *shared object*.

### The role of interdependence in the MADM model

In order to thoroughly analyze MADM across activity systems, it is also crucial to take into account another fundamental concept: *interdependence*. The concept of interdependence refers to the connection between an individual’s experiences, actions, and outcomes and those of other members within a group or a community. This concept was initially introduced by Lewin in 1948, who argued that groups form not necessarily due to similarities among members, but rather when individuals realize that their fate is dependent on the collective destiny of the group. This type of interdependence is called *“interdependence of fate.”* However, according to Lewin and subsequent authors, *“task interdependence”* is even more important for collective processes. This refers to the degree to which the goals of group members are interdependent, meaning that the success of one individual directly impacts the success of others or is even necessary for others to succeed (Lewin, 1948; Brown, 1990).

According to us, the concept of interdependence can also be translated into the MADM model to describe the relations between multiple activity systems. Indeed, interdependence can be determined by the implications that one actor’s decisions have on the achievement of others’ objectives. The higher the level of interdependence among activity systems, the greater the decisions’ implications by one subject has on the success or failure of others’ objectives.

## Human-AI integration: emphasizing the primacy of humans through activity theory

If on one hand AT helps us conceptualize MADM processes, on the other hand, it is also useful in understanding the role of humans in human-AI integration. In this regard, AT focuses on three central concepts helpful to analyze the relationship between humans and technologies (Kaptelinin and Nardi, 2006; Talamo et al., 2021). The first concept is the asymmetrical interaction between the subject and the object, meaning that the initiation and execution of actions are conceived by human subjects to fulfill their needs and reach the objects (Pickering, 1993 1995). The second concept refers to intentionality of human beings, implying that intentionality stands as an attribute unique to human subjects. Finally the last concept pertains to the mediation role of tools. Indeed, the previously mentioned asymmetrical interaction between the subject and the object can be mediated through a tool – whether a tangible artifact or an intangible entity such as ideas and procedures – enabling the subject to attain their ultimate objectives (Leont’ev, 1974, 1978). These concepts support the undisputed primacy of humans in the context of human-AI integration. Hence, within this theoretical framework, AI can be conceived as a mediation tool between human subjects and the objects of their actions. Indeed, AI may find application across diverse segments of the DM process, facilitating tasks like information gathering, analysis, criteria standardization, and even automating customer interactions (Haesevoets et al., 2021). However, it’s crucial to highlight that AI is fundamentally a tool devised, designed and employed by humans. Indeed, even if AI possesses agency,^4^ according to Kaptelinin and Nardi (2006), it detains only a kind of *delegated agency*. In fact, while AI may appear to act upon intentions, it is important to recognize that these intentions are essentially delegated to it by external entities (human beings). Indeed, as stated by Leont’ev (1974, 1978), the core locus of agency resides within human beings due to their close connection with the concept of intentionality (Stetsenko and Arievitch, 2004). This kind of agency is rooted in *need-based agency* (Kaptelinin and Nardi, 2006), entailing the fulfillment of biological and cultural needs through intention formation and subsequent action. As stated by Rose et al. (2005), only humans possess qualities such as *“self-awareness, social awareness, interpretation, intentionality, and the attribution of agency to others,”* which are not available to non-living things, such as technological systems.

From this analysis, it is evident how crucial it is to adopt a human-centered perspective for the development of AI-based systems. In fact, investigating and modeling human DM before translating it into technological development guarantees that agency is effectively delegated in accordance with human intentions. Consequently, a thorough examination of human intentions takes on paramount significance. Therefore, in this study we prioritize a human-centered viewpoint and a comprehensive understanding of human criteria and preferences to ensure the development of user-centered AI-based systems which are aligned with human internal DM models.

## Methodology

### Creating a MADM model from qualitative data

With the aim of modeling human DM and defining the MADM model to develop a user-centered AI-based system, we have structured a specific methodology. However, before delving into the details of the methodology, it is crucial to emphasize that in the perspective of designing a tool, a significant portion of attention is devoted to understanding and describing all actors who play a role in the MADM process by using descriptors of the AT model. The subsequent sections outline the three fundamental steps of our methodology.

- The first step involves the *exploration of the prospective actors of the MADM,* being or prospective users in different roles or the providers of the service itself to gather valuable data about their psychological and organizational world. This exploration—enabled by the use of *User Research* and *Strategic Organizational Counseling*—provides the collection of relevant information that serves as the basis for the subsequent modeling activity.
- The second step represents the *modeling of DM processes and activities specific to the actors in the MADM process*. This comprehensive modeling approach—carried out through *Thematic Analysis* (Braun and Clarke, 2006) and the systematization of data in the DT tools—consents to gain a holistic view of the different categories of prospective users involved.
- The final step aimed at *bridging all actors* by aligning their respective activities and DM processes in the *MADM model*. This model offers a comprehensive and holistic framework to capture the specificities and conditions that influence each actor’s DM process and impacts the creation of interobjectivity. By capturing the complexities of the MADM process, it becomes possible to develop a solution that addresses users’ unique needs and challenges.

### First step: user research and strategic organizational counseling

The initial phase employs *User Research* and *Strategic Organizational Counseling*, two methods for studying and exploring prospective users and providers.

User Research, which typically uses qualitative research methods to explore user needs in-depth, corresponds to the preliminary phase of the SDT process. During User Research, a specific kind of interview, called the *narrative interview* (Atkinson, 2002), is employed to explore the prospective users. This interview approach effectively captures the users’ perspective and comprehension of their thoughts. Through narrative interviews, researchers gather rich and insightful data in the form of personal stories, enabling individuals to share their lived experiences related to specific themes identified by the researcher. This method is chosen for its ability to comprehensively understand the opinions and motivations that influence individuals’ attitudes and behaviors. It allows for the exploration of intersubjective representations and diverse objectives, while its flexibility enables a multifocal investigation of various interests.

The other tool we adopted for data collection is the Strategic Organizational Counseling (Talamo et al., 2021; Marocco et al., 2023a,b). This methodology, developed by the *IDEaCT Social Lab* of Sapienza University, assists organizations in developing services and facilitating organizational processes necessary for the success of the service. Strategic Organizational Counseling employs dialogic sessions and psychological interview techniques to highlight the importance of organizational structure and processes in meeting the demands of potential customers. It aims at highlighting potential resources and obstacles to goal-oriented activities, analyzing the role of each actor involved within the provider’s organization. These goal-oriented sessions refine the flow of organizational DM processes, represented also in a visual format, starting from conceptual service design and concluding with external market integration. This *Organizational DM flow*,^5^ which results as the outcome of Strategic Organizational Counseling, helps create awareness on the actual role of the different parts of the organizational structure in the whole DM process behind the service delivery.

### Second step: thematic analysis for design thinking

In the second phase, narrative interviews can be analyzed and encoded using a *tool-centered coding criteria* based on the *Thematic Analysis* approach by Braun and Clarke (2006). *Thematic Analysis* is a qualitative method that involves identifying and analyzing recurring themes within a specific dataset. We adapted this procedure in the context of DT using a mixed approach that incorporates both deductive and inductive modes, guided by on-field exploration and the emergence of spontaneous data. The following steps are involved:

1. *Data collection*: relevant data is collected through semi-structured narrative interviews (first step: User Research and Strategic Organizational counseling);
2. *Transcription*: the collected data is transcribed into textual format;
3. *Familiarization with the data*: researchers gain a general understanding of the data content;
4. *Generation of categories*: main themes are identified using a theory-driven approach, referring to users’ psychological functioning areas identified by Bland (2016) for creating the Empathy Map (do, think, say, feel, hear, see, gain, pain). Sub-themes are developed through a data-driven approach, capturing the main stages of the development path used for creating the basis of the Activity Diagram (Young, 2008);
5. *Coding*: different parts of the data are assigned to the identified categories;
6. *Revision and refinement*: categories are reviewed to ensure accurate representation of the data;
7. *Themes analysis*: the identified themes and sub-themes are analyzed for integration into subsequent DT tools, such as the Empathy Map and the Activity Diagram.

Once the analysis is complete, SDT tools can be created. To create the MADM model and support the development of Multi-Actor DSS, a specific selection of tools can be chosen, including the Empathy Map (Bland, 2016), Personas (Osterwalder and Pigneur, 2013), Activity Diagram (Young, 2008), MADM flow^6^ (Marocco et al., 2023b) that is an adaptation of the User Journey Maps^7^ (Stickdorn and Schneider, 2011), and the Organizational DM flow (Marocco et al., 2023a,b) that emerges from the Strategic Organizational Counseling.

### Third step: MADM model

This last step focuses on bridging the users and providers by aligning their activities and DM processes within the MADM model. The MADM model (Marocco and Talamo, 2022), based on the third generation of AT (Engeström, 2001), offers a comprehensive and holistic framework that captures the specificities and conditions that influence each actor’s DM process and impacts the creation of interobjectivity. To address this challenge, we provide detailed instructions to integrate the different components of a single activity diagram, starting from the specific DT tools used during this process (see Marocco et al., 2023b).

**Figure 2:**
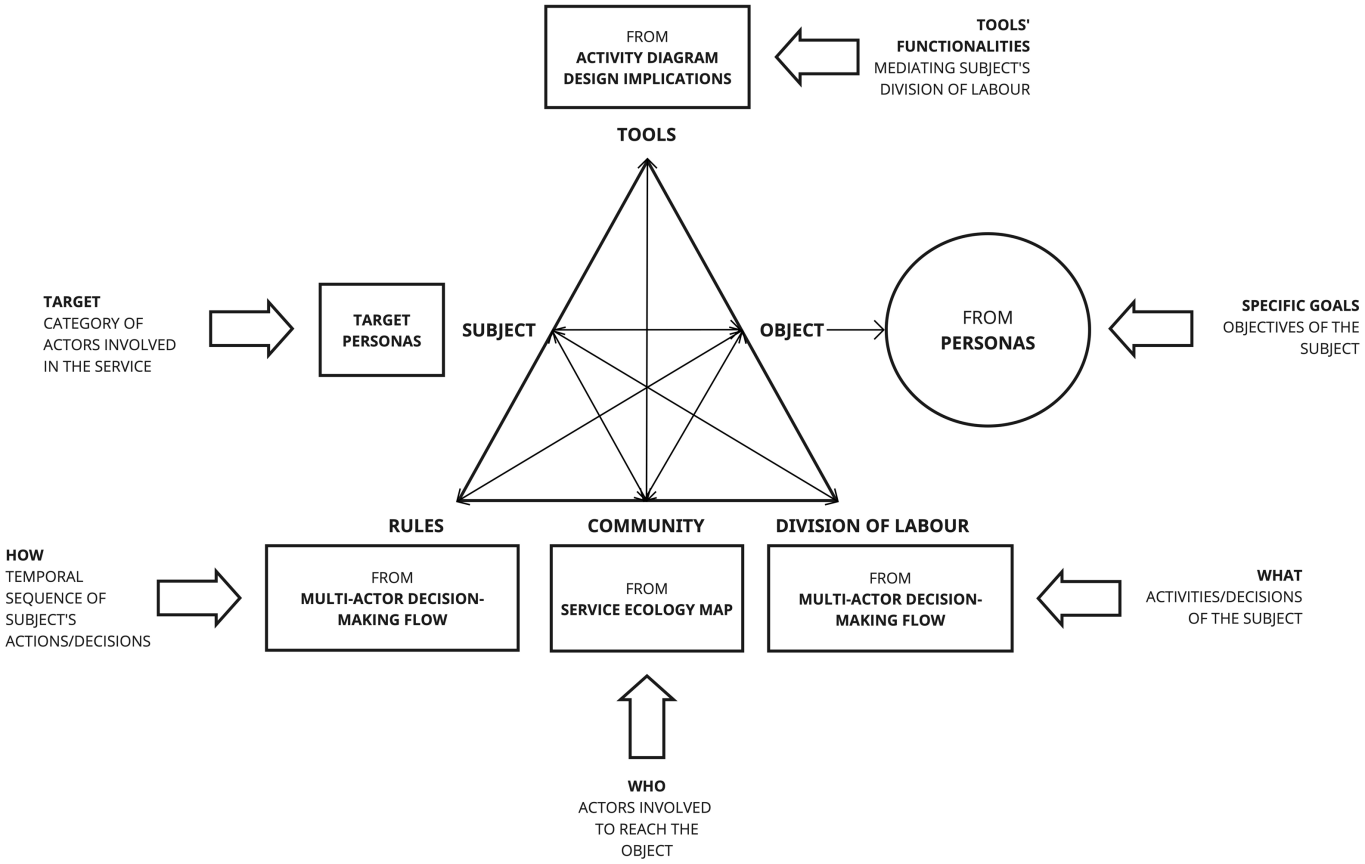
Instructions to convert design thinking tools into activity systems components.

As depicted in Figure 2, the activity system components can be effectively interpreted within the service framework, illustrating how the respective needs of the subject are addressed through the new service.

Specifically, activity systems components can be derived from the following DT tools:

- The *subject* component is derived from the target *Personas* and represents the category of actors involved in the service. Personas are archetypes of real people (Osterwalder and Pigneur, 2013), through which different target’s spheres of interest, such as goals, abilities, activities, motivations, needs, and obstacles can be explored.
- The *object* component represents the horizon towards which the specific objectives of the *Personas* are orientated.
- The *division of labor*, or the *“what,”* encapsulates the activities and decisions undertaken by the subject, drawing insights from the *MADM flow*. The *MADM Flow* (Marocco et al., 2023b) describes how Personas engage in DM and interact with one another. Concretely, it can be derived from User Journey Maps to capture the sequential actions and decisions of the Personas.
- The *rules*, or the *“how,”* originate from the *MADM flow*, delineating the temporal sequence of actions and decisions made by the subjects.
- The *community*, or the *“who,”* is extracted from the *Service Ecology Map*, spotlighting the diverse actors engaged in achieving the subjects’ objectives. Service Ecology Map (Polaine et al., 2013), consents to have a concrete representation of the complexity of the service environment and of the multiplicity of actors to involve. Indeed, Service Ecology Maps are particularly useful in the early stages of design, as they offer a means of establishing a shared overview of the work and DM space.
- The *tools* component, extracted from the analysis of the *Activity Diagram*, encompasses the functionalities of the new tool and service that mediates the division of labor of the subjects and their community, addressing the specific goals directed towards the object. The Activity Diagram, also known as *Mental Model* (Young, 2008), is employed by psychologists as a diagram of activities in which it is indicated what the user performs through the mediation of artifacts.

This conversion must be carried out for all the actors involved in the MADM process, encompassing both the users of the DSS and the organization providing the service. In this manner, these distinct activity systems will be then consolidated into the unified MADM model. To illustrate this process with a concrete example, we present below the model adapted to a specific case study within the context of organizational development.

## AHEDA case study: MADM in the field of organizational development

Our case study was offered by Mylia, a brand within The Adecco Group that focuses on training and development for individuals and companies. Specifically, our research team, which belongs to IDEaCT Social Lab (Department of Developmental and Social Processes) at Sapienza—University of Rome, collaborated with Mylia’s Design & Innovation team during the initial stages of “AHEDA” development. AHEDA is an AI-based tool for enhancing organizational behavior. Precisely, its principal aim is to assist coaches and trainers in identifying tailored profiles and development pathways for professionals. The research project behind AHEDA, which involved multidisciplinary research and collaboration with other Italian Universities, included several key activities: creating a psychological questionnaire to understand organizational behavior, developing a behavior mapping tool, and implementing an AI-based system based on a probabilistic predictor in the form of a Dynamic Bayesian Network (DBN; Catellani et al., 2021, 2022) to assist coaches and trainers in identifying the most suitable development pathway. In particular, the AHEDA psychological questionnaire investigates organizational behavior according to a model of 10 dimensions: Emotional Balance, Openness to Risk, Data Driven Mindset, Trust, Time Management, Networking, Team Building, Influence, Organizational Identity, and Fulfillment. These psychological dimensions are interconnected within a network of associative-causal relationships, serving as predictors of occupational achievement. This outcome is achieved through the application of AI techniques to identify the probabilistic causal model with the best combination of explanatory and predictive capabilities. The resulting AHEDA model forms the basis of the algorithm for searching paths to suggest based on the initial profile and desired improvements. This implies that the predictive computing model underlying AHEDA has a high predictive power of relationships between dimensions, allowing for the anticipation and exploration of the optimal development path for each coachee. The suggested paths and target profiles are valuable for the coach in designing a training program that is most effective in achieving the individual’s goals.

Our research team’s primary role was to provide consultancy support to Mylia, aligning and integrating the existing service concept with the insights obtained from the SDT process.

Due to Mylia’s requirements, the scope of the pilot experimentation was focused solely on the individual development path (coaching) in the B2B scenario, rather than on training or on the B2C scenario. Consequently, our research has primarily concentrated on the coaching target.

**Figure 3:**
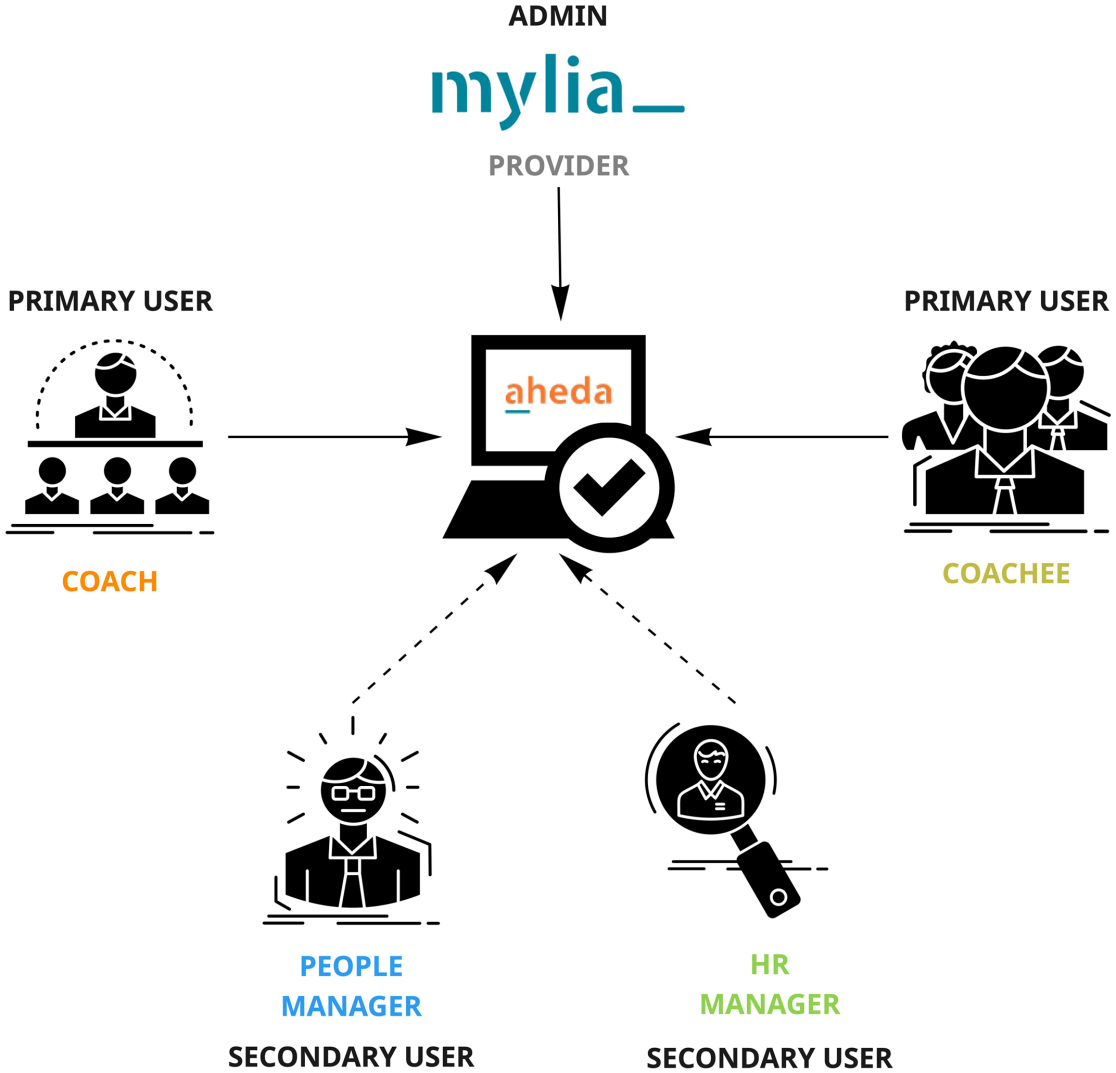
Users of AHEDA DSS.

Starting from this general objective, we realized during our meetings participation that MADM was under discussion. It became evident that it was necessary to model the activities of all the actors involved in the process, who would also be prospective users of the platform. More specifically, we identified five crucial categories of actors to explore, distinguishing them between *primary users* (coaches—partners of the provider organization—and coachees), *secondary users* (HR and people managers) and the *service provider/admin* (Mylia). Furthermore, our work takes on a fundamental role in determining which of users’ activities could be supported by AI and which could not, with the aim of designing not only AI-based activities, but the AHEDA service as a whole (see Figure 3).

### From service design thinking to the third generation of activity theory

Starting from the SDT process, we created five activity systems—which include the provider organization and the other four categories of actors. For each of them, the various components are described below in detail, showing how the tool’s functionalities align with each actor’s objectives. From these activity systems, following the principles of third-generation AT, we developed the MADM model, where the five activity systems were put together in interaction. For the MADM model, a higher-level analysis was conducted, capturing the interdependence of relationships between all the activity systems and the way each activity, conducted by one actor within the service, is crucial for the achievement of the objectives of the others and consequently fundamental for the continuation of the service. Furthermore, we have outlined which activities can be mediated by AI and which cannot in the overall context.

#### Activity system of Mylia

**Figure 4:**
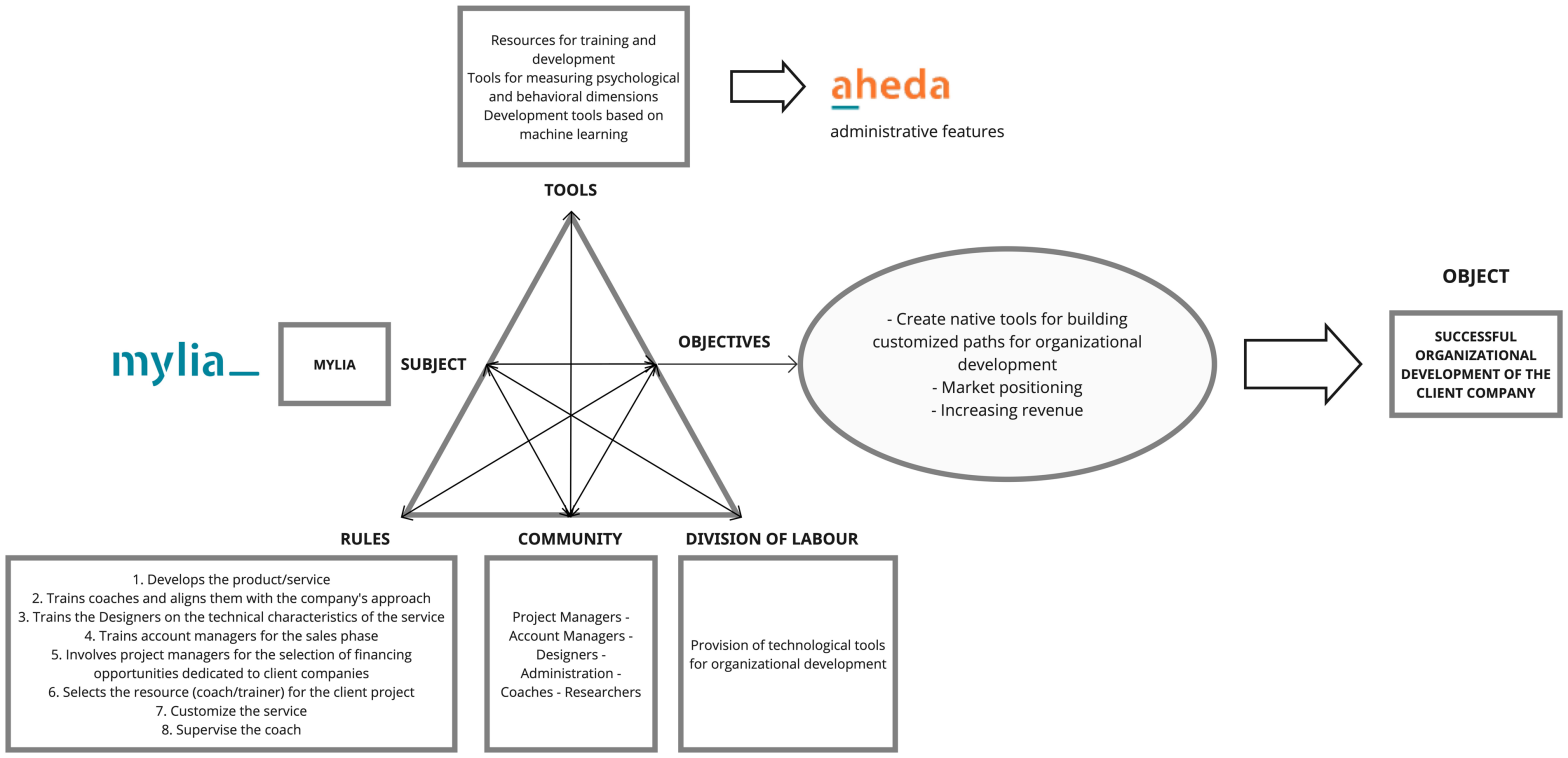
Activity system of Mylia.

The main *objectives* of MYLIA, as the provider organization, are directed to create customized tools for organizational development, enhance market positioning, and increase revenue. Based on these objectives, the *object* that MYLIA corresponds to achieving is the successful organizational development of the client company. The attainment of the object is supported by a *division of labor* in which MYLIA provides technological tools for organizational development, that is the main mission of Mylia organization. The outlined *rules* specify the temporal sequence of actions necessary to achieve the objectives, starting with the development of the product/service and progressing to the training of coaches and designers, preparing account managers for the sales phase, involving project managers for the selection of financing opportunities, selecting the resource (coach) for the client project, following all the customization phase of the service, and finally supervise the initial work of the Coach. These activities involve a *community* of actors, including Project Managers, Account Managers, Designers, Administration, Coaches, and Researchers. Mylia’s *tools* encompass resources for training and development, tools for measuring psychological and behavioral dimensions, and machine learning-based development tools. Among these tools there is AHEDA, where internal staff have access to all the administrative features (see Figure 4).

#### Activity system of the coach

**Figure 5:**
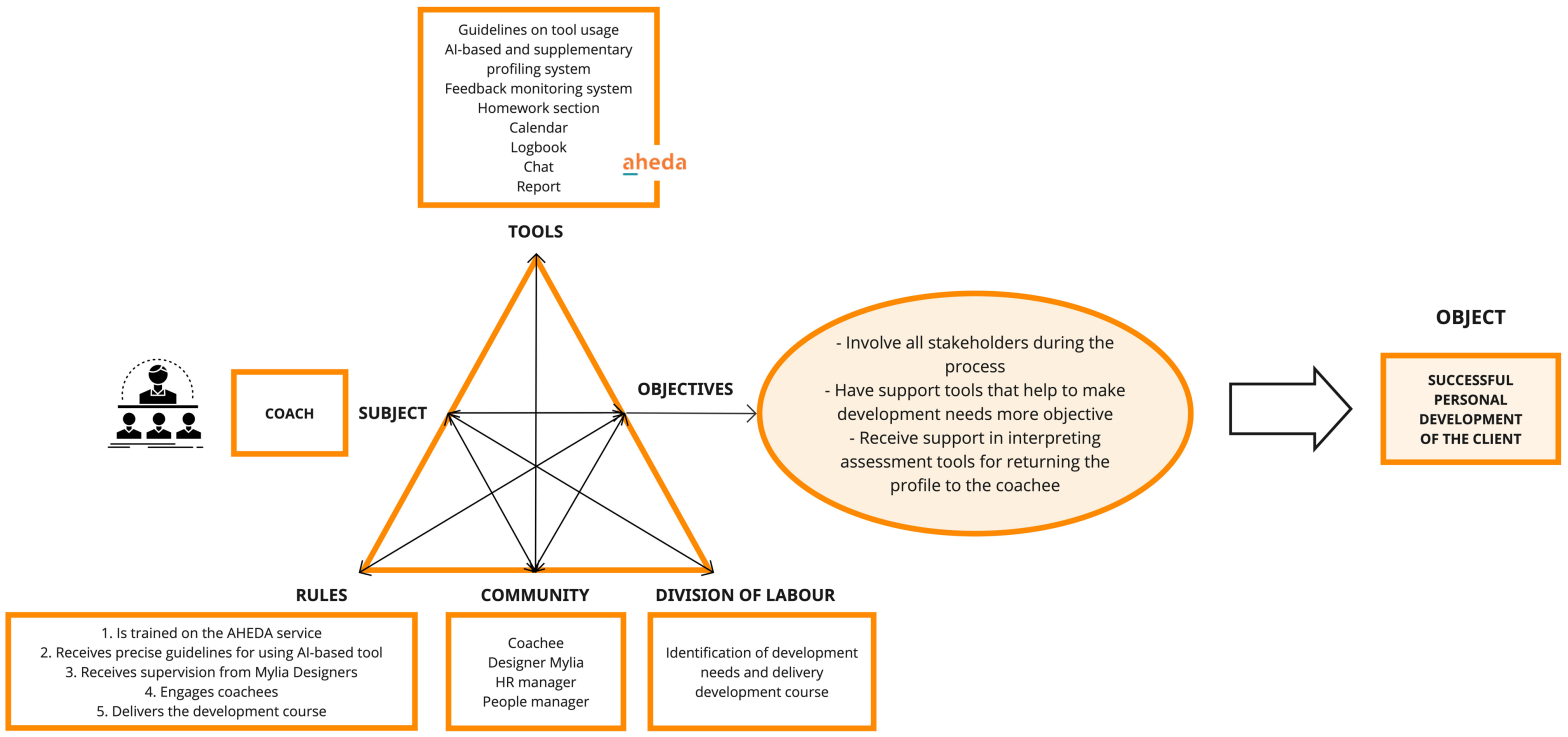
Activity system of the coach.

The primary *objectives* of the COACH include involving all relevant stakeholders, using support tools to enhance the objectivity of development needs, and receiving assistance in interpreting assessment tools for the coachees’ benefit. Respectively, these objectives are fulfilled through some functionalities of the AHEDA tool. Specifically, the profiling system, empowered by AI, assists coaches in acquiring an objective and holistic understanding of the coachee’s development needs through a quantitative survey run through a questionnaire investigating psychological dimensions such as emotional balance, networking, influence and more. This information is further integrated by a supplementary profiling tool, derived from the SDT process, that aids coaches in selecting the most suitable target profile and development path based on the recommendations generated by the AI system. Sample questions of the supplementary profiling system include the current role in the company, work experience duration, frequency of job changes, interests, values, motivation levels, and availability for development activities. The integration of both tools generates profiles encompassing both qualitative and quantitative aspects of the coachee. This integrated approach empowers the coach to make informed decisions when selecting the most appropriate development path for the coachee’s journey. Moreover, the comprehensive manual enables him/her to correctly interpret the profiling results and provide valuable feedback to the coachee; while the feedback system plays a crucial role in fostering collaboration and shared understanding among all stakeholders involved in the development journey. The *object* of the COACH is to achieve successful personal development outcomes for clients, which signifies the effective fulfillment of its job. This is achieved through a well-defined *division of labor* that involves the identification of development needs and the delivery of a tailored development path. The *rules* regulate that the COACH first undergoes comprehensive training on the AHEDA service. Then, he receives precise guidelines for the proper utilization of the AI-based tool, which serves as a crucial asset in the development process. Subsequently, ongoing supervision and guidance from Mylia Designers contribute to his/her own work effectiveness. The *community* involved in his/her division of labor includes Mylia Designers, and coachees, HR managers, and People managers from the client company (see Figure 5).

#### Activity system of the coachee

**Figure 6:**
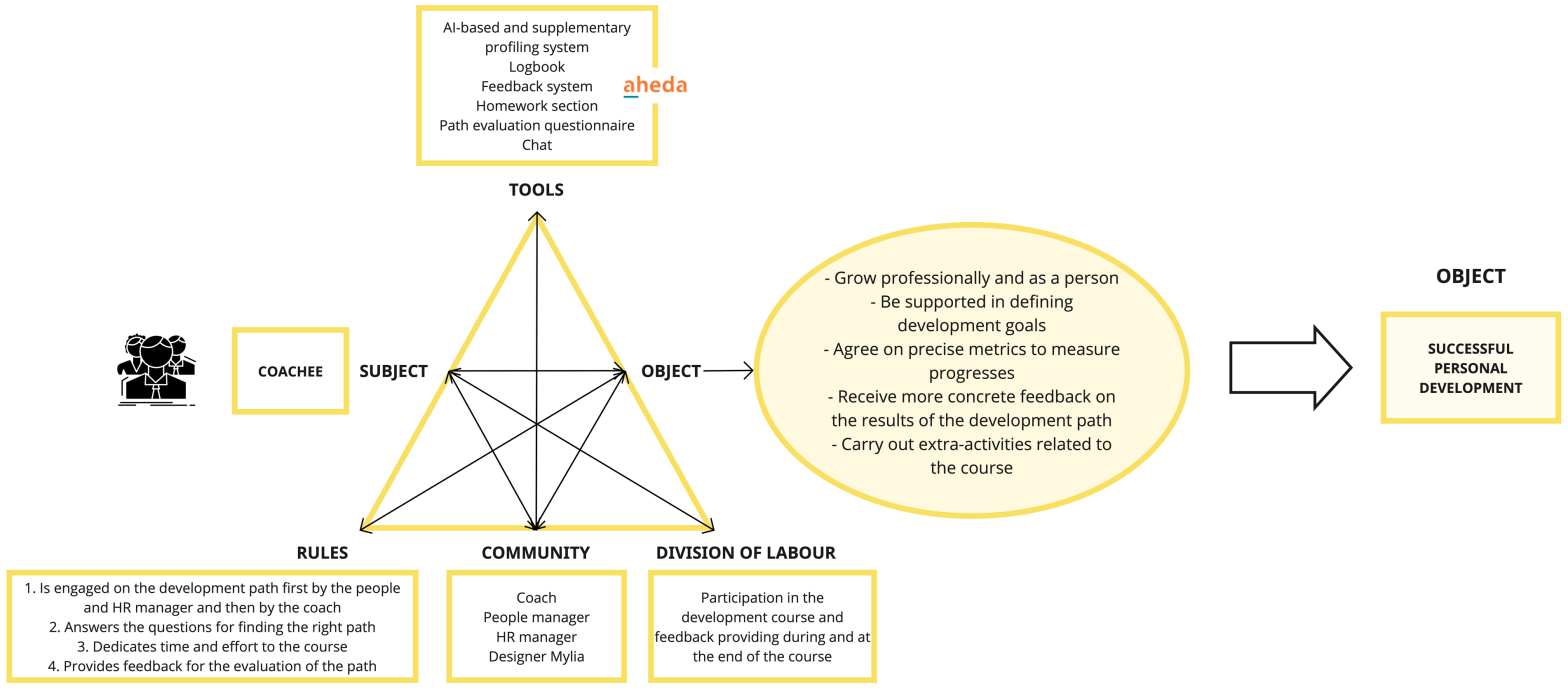
Activity system of the coachee.

The COACHEE’s aspiration encompasses multiple *objectives*, including advancing professionally and personally, receiving assistance in defining development goals, collaboratively determining precise metrics for tracking progress, acquiring more concrete feedback on the outcomes of the development journey, and engaging in supplementary activities that complement the growth path. The coachee is provided with various tools’ functionalities to reach his/her goals. The AI-based profiling system offers a deeper understanding of his/her professional and personal profile, guiding him/her in defining his/her development goals. The path evaluation questionnaire objectively assesses progress and identifies areas of improvement. The feedback system ensures regular and structured measurements, enabling his/her to assess progress, and receive more concrete insights on the results of his/her development path. The homework section promotes active learning through specific tasks outside the coaching sessions. The ultimate *object* of the COACHEE is to foster successful personal development, aligning with one of its core motivations. This achievement is realized through a well-defined *division of labor*, in which he/she takes part actively, providing ongoing feedback throughout the course’s duration and upon its completion. Guided by specific *rules*, the COACHEE operates within a specific temporal sequence of actions that includes: the initial engagement initiated by the manager, followed by continued guidance from the coach; responsiveness to essential inquiries aimed at identifying the most suitable developmental trajectory; dedication of time and effort to the prescribed course; and the contribution of valuable feedback to facilitate comprehensive path evaluation. The *community* of the COACHEE includes People Managers and HR Managers from his/her organization and Coaches and Designers from Mylia (see Figure 6).

#### Activity system of the HR manager

**Figure 7:**
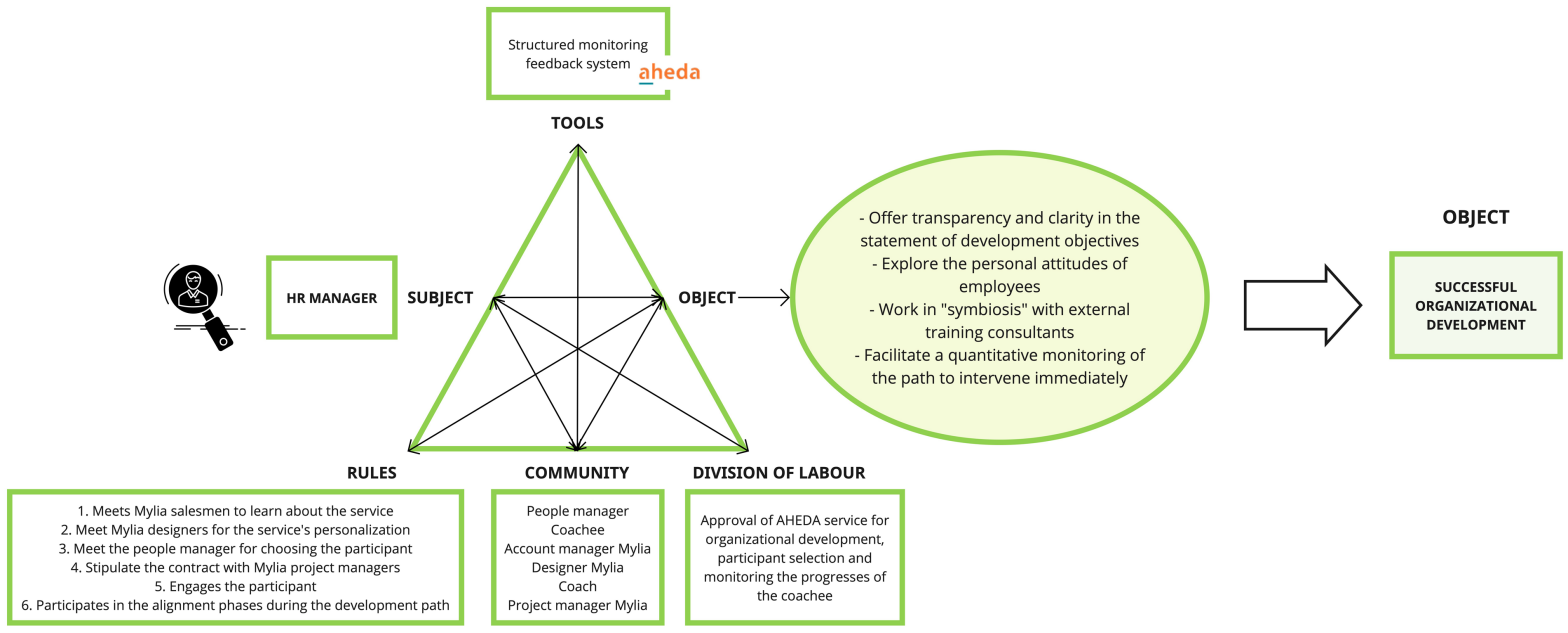
Activity system of the HR manager.

The HR MANAGER is characterized by the following set of key *objectives*, including providing transparency and clarity in articulating development objectives, exploring employees’ personal attitudes to enhance understanding, establishing a harmonious partnership with external training consultants, and enabling quantitative monitoring for immediate intervention. The main AHEDA’s functionality designed to achieve the HR MANAGER’s goals is the structured monitoring feedback system. This feature allows for a systematic and quantitative evaluation of the progress, providing valuable insights for interventions and support when needed. The *object* of the HR MANAGER strives to achieve a successful organizational development. This requires a strategic division of labor, characterized by the approval of AHEDA service for organizational development, and the monitoring of the cochee’s progress in his/her development journey. The *rules* establish this sequence of actions: first, engagement with Mylia’s sales team to gain familiarity with the service, then collaborating with Mylia designers for service personalization, consulting the People manager to select the suitable participant, finalizing a contractual agreement with Mylia project managers, and actively participating in alignment phases throughout the coachee’s developmental journey. The division of labor of the HR MANAGER involves a *community* of key actors: the People Manager and the coachee from his/her organization, the coach, and the Account Manager, Designer, and Project Manager from Mylia (see Figure 7).

#### Activity system of the people manager

The PEOPLE MANAGER possesses a set of clear and decisive *objectives* which are central to his/her operation, such as systematizing the aggregation of training needs, defining measurable objectives to assess post-course improvements, raising employees’ awareness of their development needs, and equipping the ability to address and rectify errors or challenges encountered during the development path.

**Figure 8:**
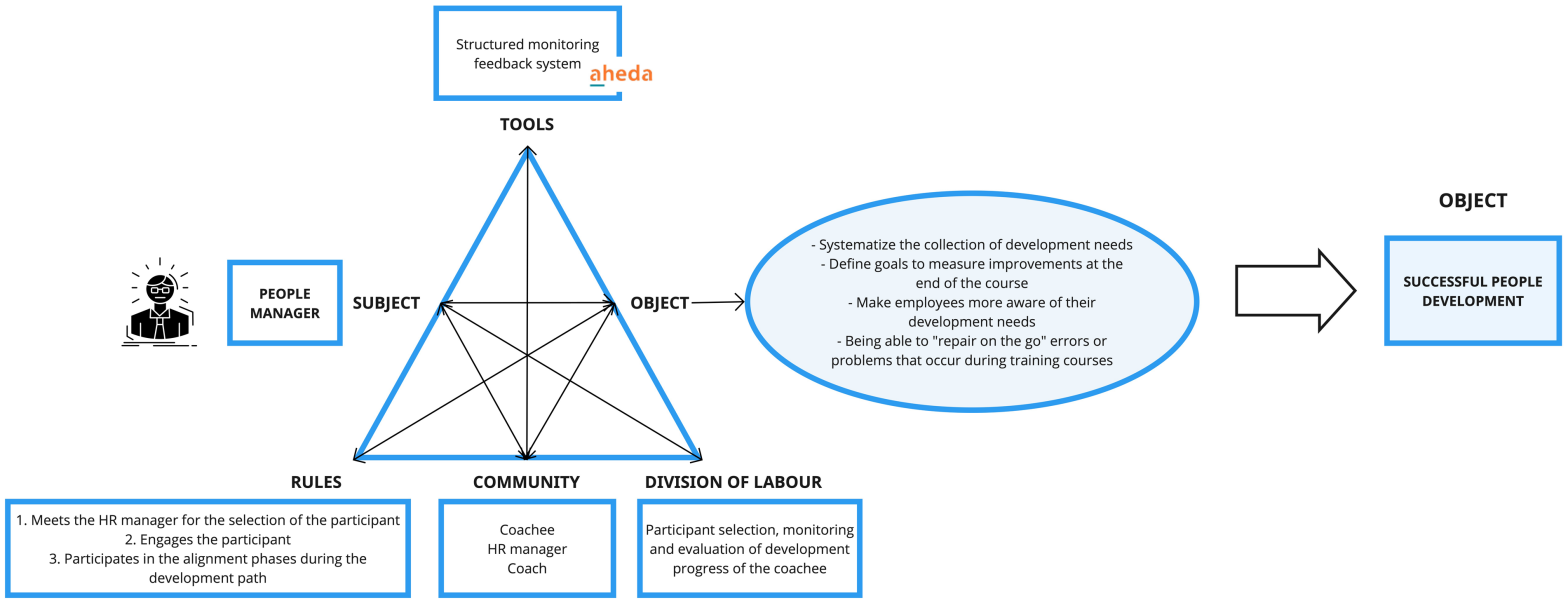
Activity system of the people manager.

To address some of his/her needs, the PEOPLE MANAGER uses as a specific *tool*’s functionality, the structured monitoring feedback system, which allows for organized and methodical tracking of the coachee’s advancement, gathering feedback, appraising the training program’s effectiveness, and enabling timely intervention when necessary. The *object* of the PEOPLE MANAGER is directed to attain successful personal development, and it is guided by a strategic division of labor, including selecting participants for the development journey, and vigilantly monitoring and evaluating the coachee’s progress. The guiding *rules* shaping the actions of the PEOPLE MANAGER are ordered as follows. The process begins with collaborative engagement alongside the HR Manager in the participant selection phase. Following this, the PEOPLE MANAGER actively involves the chosen participant, fostering their ongoing engagement. This engagement is maintained throughout alignment phases, ensuring continuous and active participation during the coachee’s developmental journey. The key actors that constitute the *community* of the PEOPLE MANAGER are the coachee, the coach and the HR Manager from his/her organization (see Figure 8).

#### AHEDA MADM model

**Figure 9:**
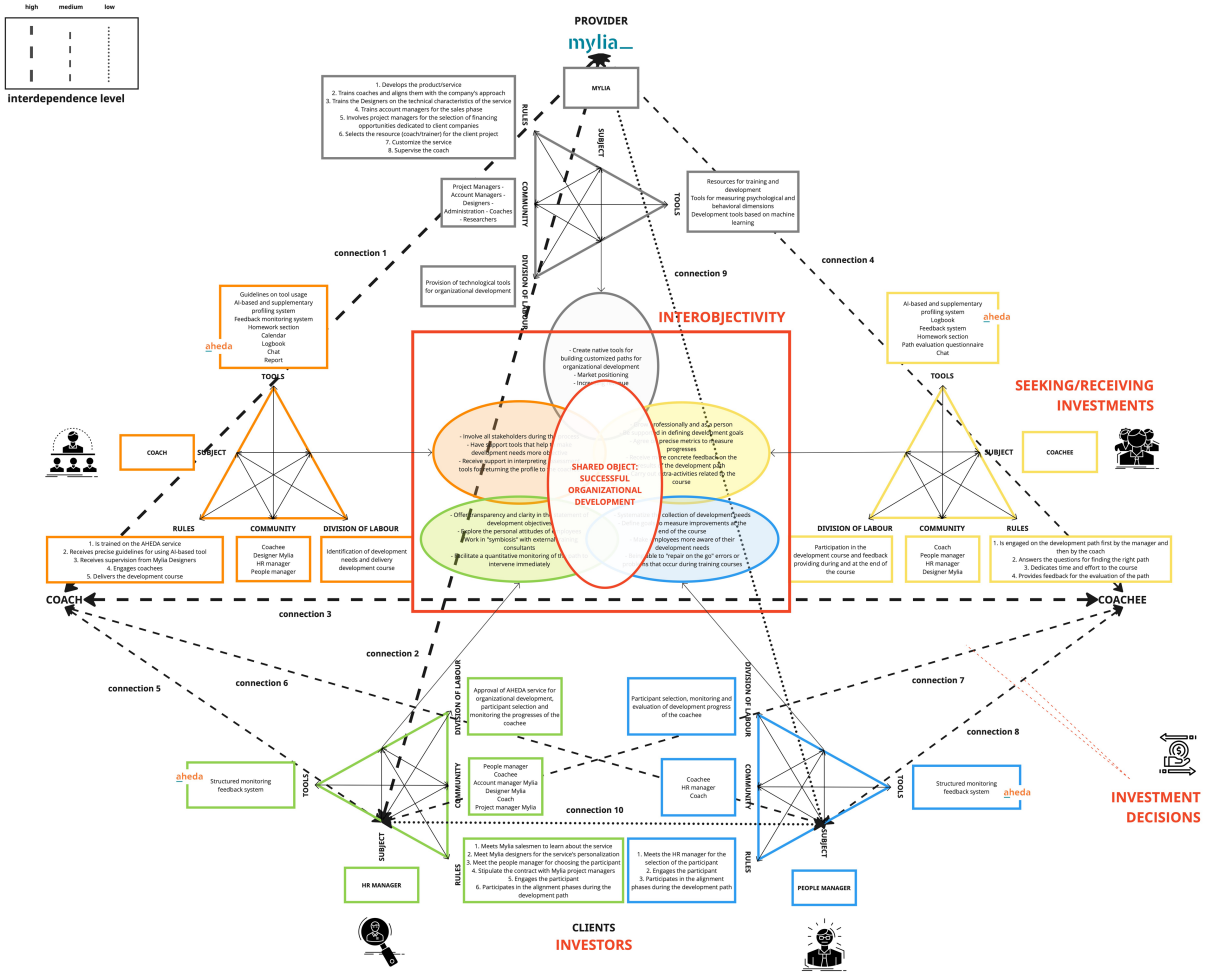
AHEDA MADM model.

This model (Figure 9) encompasses the five previously presented activity systems within a unified framework. These activity systems include those of Mylia (the provider organization), the coach, the coachee, the HR manager, and the People manager. These activity systems engage in mutual interaction, each stemming from distinct yet potentially aligning objectives that converge towards a shared object. This shared object, while originating from diverse motivations, ultimately finds its common ground in organizational development.

Moreover, each activity system is described in terms of specific components, highlighting the key interdependent aspects associated with each actor in the system. The model also visually depicts the connections between the various activity systems, represented by lines of varying thickness (see legend within Figure 9). Connections’ thickness is based on the level of decisional interdependence that occurs within the perimeter stated by the activity system’s *“division of labor”* and *“rules”* on the achievement of other activity systems’ *“objectives.”* Hence, the depth of these relationships can be determined by the impact that the decisions of the subjects have on achieving the goal, and how crucial this goal is for the continuation of the service. We have classified these relationships into high, medium, and low tiers:

- *High level of interdependence*: if the failure of one subject to fulfill their division of labor or to respect their rules implies the failure to achieve the objective of another subject, resulting in the interruption or failure of the service.
- *Medium level of interdependence*: if the failure of one subject to fulfill their division of labor or to respect their rules implies the failure to achieve the objective of another subject, significantly compromising the success of the service, but still ensuring the continuation of the core focus of the service.
- *Low level of interdependence*: if the failure of one subject to fulfill their division of labor or to respect their rules implies the failure to achieve the objective of another subject, leading to user dissatisfaction but still ensuring the continuation of the service.

Below, we provide concrete examples from the AHEDA MADM model:

1. A *high level of interdependence* can be observed between the coach and Mylia (connection 1). The coach, which seeks support tools to enhance the objectivity of development needs, necessitates to be trained by Mylia on AHEDA tool usage and be selected by Mylia for work projects (division of labor). At the same time, Mylia depends on your decision to undergo training to offer a qualified service.
2. It is also evident that Mylia has a *high level of interdependence* with the HR manager since HR’s approval (division of labor) directly impacts Mylia’s objective to sell the service and increase revenue (connection 2). On the other hand, the HR manager’s objective to facilitate a quantitative monitoring of the development path depends on the creation and provision of Mylia’s technological tool (division of labor).
3. A *high level of interdependence* is evident between the coachee, aspiring for professional and personal growth, and the coach who, as part of a division of labor, undertakes the task of identifying developmental needs and delivering developmental paths (connection 3).
4. A *medium level of interdependence* can be observed between the coachee, seeking support in defining his/her development needs, and Mylia’s division of labor, responsible for providing AHEDA tool, capable of profiling employees and facilitating the identification of the most suitable development paths (connection 4).
5. A *medium level of interdependence* can be observed between the coach and the People and HR managers (connection 5, 6). Indeed, the coach’s objective to involve all stakeholders during the process in order to work with a more self-aware coachee and to foster an attitude of receptiveness to change within the coachee’s surrounding ecosystem, necessitates the active involvement of the People manager and HR manager. Their role in monitoring the coachee’s progress (division of labor) and their rules to engage participants before the development journey has therefore a direct impact on the coach’s objective.
6. Additionally, the coachee’s objective of receiving more specific feedback on the outcomes of the development path is contingent upon the division of labor of the HR and the people managers, who are responsible for offering feedback throughout the course and upon its completion. For this reason, this relationship is also based on a *medium level of interdependence* (connection 7, 8).
7. There exists a *low level of interdependence* between Mylia and the People Manager (connection 9). In fact, the People Manager, who aims to address errors or issues that arise during development courses in real-time, relies on the functionality of the structured monitoring and feedback system provided by the Mylia tool. Nevertheless, it is primarily the responsibility of the coach and the coachee to include the People Manager’s in the development path.
8. In conclusion, a *low level of interdependence* is observed between HR and the People Manager (connection 10), both tasked with monitoring the coachee’s progress, which aligns with their respective goals. In fact, the HR manager aims to enable quantitative monitoring of the path for immediate intervention, while the People Manager seeks the capacity to address errors or issues that arise during training courses in real-time. Nevertheless, even though the contributions of both facilitate the possibility of obtaining immediate feedback, the achievement of these objectives can also be pursued individually, albeit with lesser effectiveness.

From these examples, it becomes evident that each activity system plays a crucial role in enabling others to accomplish their specific objectives. This is because some components of the activity system, the *division of labor* or *rules*, directly impact the objectives of other activity systems. This implies that each activity, directed by the subject, relies on the decision of the subject to implement it or not. This is why we refer to interdependence not only in terms of tasks but also decisions. Consequently, every decision is important as part of a single flow that enables the attainment of specific objectives and the realization of the shared object. This shared horizon—the successful organizational development—is partially shared among all the activity systems, each contributing with its own role towards the creation of interobjectivity.

**Figure 10:**
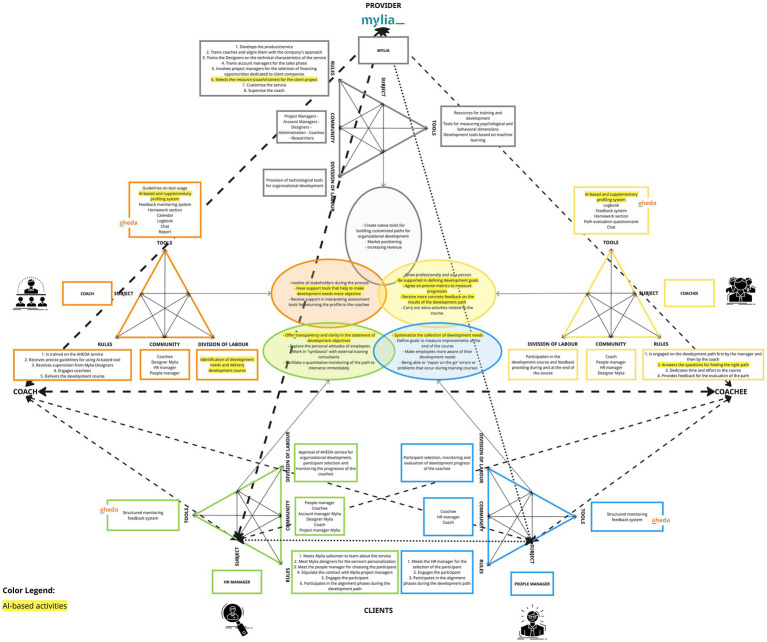
The role of AI within AHEDA MADM model.

Moreover, this type of analysis has allowed us to understand how crucial human contribution is for the success of a technological service. Indeed, by conducting an analysis of the MADM model, we can observe that AI-mediated activities and functionalities are in the minority compared to those not mediated by AI. Precisely the activities and functionalities (highlighted in yellow in Figure 10) are aimed at:

- Identifying development needs and delivering tailored development course;
- Offering transparency and clarity in the statement of development objectives;
- Making development needs more objective;
- Measuring progresses through precise metrics;
- Giving more concrete feedback on the results of the development path;
- Systematizing the collection of development needs;
- Selecting the most suitable resource (coach/trainer) for the client project.

**Figure 11:**
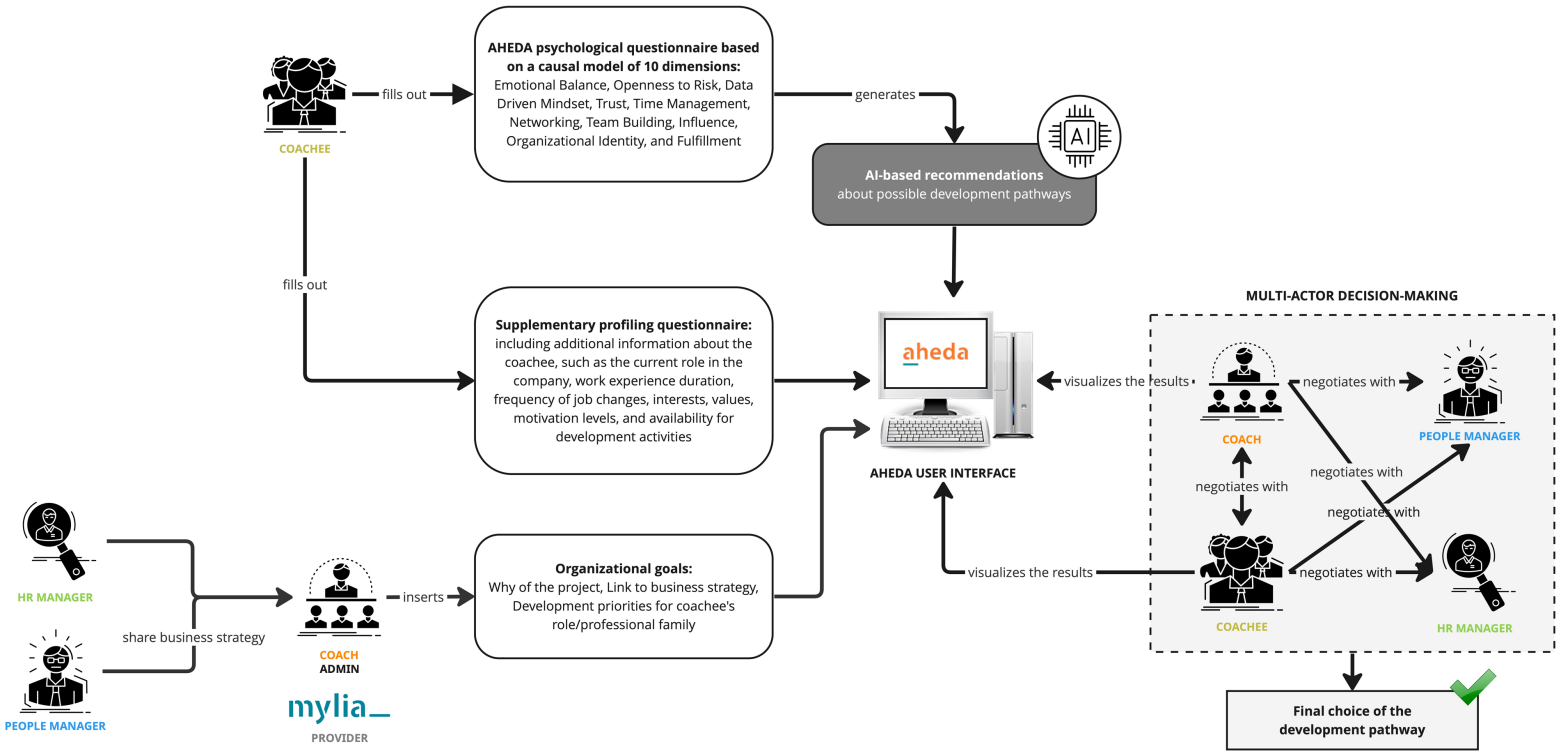
An exampling use case scenario of AHEDA DSS.

To provide a concrete example of AHEDA’s functioning, we present a use case scenario (Figure 11) illustrating one of the main AI-supported MADM processes, which is related to the identification of development pathways. In this scenario, the five users of the AHEDA DSS and the input from AI are involved. Precisely, HR and People Managers align their business strategy with the coach and Mylia as service provider. The coach then inputs organizational goals into AHEDA, specifying the Project Why, the link to the business strategy, and the development priorities for the coachee’s role/professional family. Subsequently, the coachee completes two questionnaires: the AHEDA psychological questionnaire, which encompasses 10 psychological dimensions (Emotional Balance, Openness to Risk, Data Driven Mindset, Trust, Time Management, Networking, Team Building, Influence, Organizational Identity, and Fulfillment), and a supplementary profiling questionnaire derived from the SDT process. This supplementary questionnaire provides additional information about the coachee, including their current role in the company, duration of work experience, frequency of job changes, interests, values, motivation levels, and availability for development activities.

The results from the psychological questionnaire generate AI-based recommendations for potential suitable development pathways, while the supplementary profiling questionnaire supports the coach in the final DM by offering additional insights into the coachee’s profile. Additionally, the identification of the development pathway is supported by data obtained from the initial meeting with HR and People Managers regarding the definition of organizational goals. Ultimately, the coach engages in negotiations with the other key decision-makers (coachee, HR manager, and People manager), proposing his choice and negotiating it to arrive at the final decision regarding the most suitable development path for the coachee.

## Conclusion

In the landscape of DM processes, the integration of AI, especially machine learning, has introduced transformative potential, with AI gradually assuming responsibilities that were traditionally attributed to humans (Vincent, 2021). However, while AI promises enhanced efficiency and accuracy, its adoption is met with resistance in many organizational settings, since individuals still strongly prefer human intervention (Haesevoets et al., 2021). The barriers to AI acceptance encompass psychological factors and other trust-related issues (Cao et al., 2021; Leyer and Schneider, 2021; Mahmud et al., 2023). Recognizing the significance of trust in AI systems (Shin, 2021), recent studies emphasize the importance of user-centered design (Shin, 2021). To this aim, our study bridges SDT and AT (Leont’ev, 1974, 1978; Engeström, 1987, 2001) to create practical guidelines for the user centered design of Multi-Actor AI-based DSS. In particular, we provide practical instructions for the creation of a MADM model within the field of IHC, specifically in the context of organizational development. According to us, the creation of a MADM model, which includes the fundamental elements of the entire SDT process, can be extremely valuable in the development of a user-centered Multi-Actor DSS as it provides a detailed and comprehensive overview of the specifics of the different actors involved in the complex MADM process. First of all, it describes the social context in which technology will be implemented, defining the interactions and relationships among different actors. This socially contextualized approach offers an in-depth analysis of the environment in which the tool will be introduced for mediating already established social practices. Moreover, it highlights the way technological functionalities are tailored to address the unique requirements of each actor within the IHC context, highlighting how technology is intentionally designed to serve the needs of its users, and reinforcing its role as a tool in support of human endeavors. Hence, this MADM model is created through the qualitative study of complex human activity as a unit of analysis. Nevertheless, it holds the potential for further enhancement through the application of quantitative methods to explore its diverse dimensions more extensively. For instance, during our study, we were able to identify which aspects could be assessed using quantitative questionnaires.

Additionally, this kind of analysis provides crucial insights into the importance of human contribution in designing technological systems. From this study, it is evident that AI is not a comprehensive solution but rather addresses specific tasks or functions within the broader context of a service or a system. Indeed, AI is integrated as a component within the service, providing functionalities that assist and enhance certain aspects of human activity. Hence, while AI can automate specific tasks, it does not operate in isolation; rather it is integrated into a system heavily influenced by human action. This ecosystem comprises interdependent activities and decisions among multiple actors, negotiations, communication exchanges, and steps that are not mediated by technology but still need to be defined as touchpoints for the functioning and overall success of the service. Therefore, trust in such AI-based systems is not solely based on the components of AI but on the overall reliability of the entire service.

Users place their trust in the service as a whole, including how AI is integrated, how it interacts with human users, and how effectively the service supports the achievement of their specific goals and objectives. Consequently, we believe that building trust in AI-based systems requires a holistic approach that considers the entire ecosystem of services and its capacity to effectively meet user needs. In this perspective, the MADM model represents an effective holistic tool to keep on board during the design process all the specificities of the decision system, including the intricate interdependencies and the human and artificial contributions that together collaborate for the success of the service. For this reason, this tool can be crucial for both developers and designers to acknowledge these factors before embarking on technology development.

## Data availability statement

The raw data supporting the conclusions of this article will be made available by the authors, without undue reservation.

## Ethics statement

The study was approved by the Ethical Commission of the Sapienza University of Rome (CERT_18D65558A04). Participation was voluntary, without any compensation. The participants provided their written informed consent to particpiate in this study.

## Author contributions

SM: Conceptualization, Data curation, Methodology, Writing – original draft, Writing – review & editing. AT: Conceptualization, Supervision, Writing – review & editing. FQ: Project administration, Supervision, Writing – review & editing.

## Footnotes

^1^Rules: refer to sets of conditions, whether formal or informal, that determine how and why individuals can act and are influenced by social conditioning (Engeström, 1987).

^2^Division of labor: involves the distribution of roles and tasks among a community of workers (Engeström, 1987).

^3^Community: is a new dimension where groups of activities and teams of workers are situated and can be analyzed (Engeström, 1987).

^4^Agency: *“the ability to act in the sense of producing effects according to an intention”* (Kaptelinin and Nardi, 2006, p. 33).

^5^Organizational DM flow: this tool shows how the DM processes, linked to various professional families and actors of the provider organization, need to be governed to ensure the success of the service (Marocco et al., 2023a).

^6^MADM flow: this tool refers to a comprehensive representation of how Personas engage in DM processes and interact with one another (Marocco et al., 2023b).

^7^User Journey Map: a tool that provides a vivid, concise, structured, and timely visualization of the User Experience of a service (Stickdorn and Schneider, 2011).
